# Associations between Pre-Bariatric High-Sensitivity C-Reactive Protein and Post-Surgery Outcomes

**DOI:** 10.3390/diagnostics11040721

**Published:** 2021-04-18

**Authors:** Tannaz Jamialahmadi, Mohsen Nematy, Simona Bo, Valentina Ponzo, Ali Jangjoo, Ladan Goshayeshi, Aida Tasbandi, Nikita G. Nikiforov, Amirhossein Sahebkar

**Affiliations:** 1 Department of Nutrition, Faculty of Medicine, Mashhad University of Medical Sciences, Mashhad 91779-48564, Iran; 2 Department of Medical Sciences, AOU Città della Salute e della Scienza di Torino, University of Turin, 10126 Torino, Italy; 3 Surgical Oncology Research Center, Imam Reza Hospital, Faculty of Medicine, Mashhad University of Medical Sciences, Mashhad 91779-48564, Iran; 4 Department of Gastroenterology and Hepatology, Faculty of Medicine, Mashhad University of Medical Sciences, Mashhad 91779-48564, Iran; 5 Gastroenterology and Hepatology Research Center, Mashhad University of Medical Sciences, Mashhad 91779-48564, Iran; 6 Applied Biomedical Research Center, Mashhad University of Medical Sciences, Mashhad 91779-48564, Iran; 7 Laboratory of Cellular and Molecular Pathology of Cardiovascular System, Institute of Human Morphology, 3 Tsyurupa Street, 117418 Moscow, Russia; 8 Laboratory of Medical Genetics, Institute of Experimental Cardiology, National Medical Research Center of Cardiology, 121552 Moscow, Russia; 9 Biotechnology Research Center, Pharmaceutical Technology Institute, Mashhad University of Medical Sciences, Mashhad 91779-48564, Iran; 10 School of Pharmacy, Mashhad University of Medical Sciences, Mashhad 91779-48954, Iran; 11 Department of Medical Biotechnology, School of Medicine, Mashhad University of Medical Sciences, Mashhad P.O. Box 91779-48564, Iran

**Keywords:** inflammation, bariatric surgery, steatohepatitis, fatty liver disease

## Abstract

Background: Obesity is a chronic inflammatory condition associated with increased circulating levels of C-reactive protein (CRP). Bariatric surgery has been reported to be effective in improving both inflammatory and liver status. Our aims were to elucidate the relationships between pre-surgery high sensitivity-CRP (hs-CRP) values and post-surgery weight loss and liver steatosis and fibrosis in patients with severe obesity undergoing Roux-en-Y gastric bypass. Methods: We conducted an observational prospective study on 90 individuals with morbid obesity, who underwent gastric bypass. Anthropometric indices, laboratory assessment (lipid panel, glycemic status, liver enzymes, and hs-CRP), liver stiffness and steatosis were evaluated at baseline and 6-months after surgery. Results: There was a significant post-surgery reduction in all the anthropometric variables, with an average weight loss of 33.93 ± 11.79 kg; the mean percentage of total weight loss (TWL) was 27.96 ± 6.43%. Liver elasticity was significantly reduced (from 6.1 ± 1.25 to 5.42 ± 1.52 kPa; *p* = 0.002), as well as liver aminotransferases, nonalcoholic fatty liver disease fibrosis score (NFS) and the grade of steatosis. Serum hs-CRP levels significantly reduced (from 9.26 ± 8.45 to 3.29 ± 4.41 mg/L; *p* < 0.001). The correlations between hs-CRP levels and liver fibrosis (elastography), steatosis (ultrasonography), fibrosis-4 index, NFS, and surgery success rate were not significant. Regression analyses showed that serum hs-CRP levels were not predictive of liver status and success rate after surgery in both unadjusted and adjusted models. Conclusions: In patients with morbid obesity, bariatric surgery caused a significant decrease in hs-CRP levels, liver stiffness and steatosis. Baseline hs-CRP values did not predict the weight-loss success rate and post-surgery liver status.

## 1. Introduction

Obesity has been widely recognized as an inflammatory condition [1,2,3,4]. Increased levels of C-reactive protein (CRP), a serum acute-phase reactant produced by the liver, and other proinflammatory cytokines secreted by the adipose tissue such as interleukin-6 (IL-6) and tumor necrosis factor-α (TNF-α), have been reported in patients with obesity [5,6,7,8]. This low-grade chronic inflammatory state has been associated with insulin resistance, endothelial dysfunction, higher risk of cardiovascular diseases and mortality [9,10,11,12].

Bariatric surgery is reported to be effective both in the reduction of adipose tissue mass [13,14] and in the improvement of the obesity-associated co-morbidities, such as type 2 diabetes mellitus (T2DM), arterial hypertension, hyperlipidemia, obstructive sleep apnea [15], non-alcoholic fatty liver disease (NAFLD) and steatohepatitis (NASH) [16,17,18,19]. Additionally, several studies have shown that bariatric surgery improved inflammatory status and reduced CRP values regardless of weight loss changes [20,21,22,23,24,25,26,27,28,29,30,31,32]. This implies that interactions between a collection of factors can regulate inflammation.

The role of CRP values in relation to bariatric surgery is now considered relevant, as post-surgery increment in serum CRP concentrations is a useful and cost-effective marker to identify early postoperative leak and complications [33,34]. Therefore, CRP can be used as a monitoring biomarker and also as a clinical parameter for hospital discharge [35,36]. On the other hand, only few and contrasting data are available about the predictive role of pre-bariatric CRP values on post-bariatric outcomes. Pre-surgery low CRP concentrations have been associated with post-bariatric weight loss [37]. Other authors found the opposite, i.e., the higher the pre-surgery CRP levels, the greater the reduction of body fat [38] and the possibility of T2DM remission [39], suggesting pre-bariatric CRP value as a valuable predictor of surgery favorable outcomes. To the best of our knowledge, no data was available in literature about the association of this marker with NAFLD, one of the more frequent comorbidities of obesity.

The purpose of the present observational prospective study was therefore to elucidate the relationships between pre-surgery high sensitivity-CRP (hs-CRP) values and post-surgery weight loss and liver steatosis and fibrosis in patients with severe obesity undergoing to a Roux-en-Y gastric bypass.

## 2. Material and Methods

The study was conducted from December 2016 to September 2017 in 90 morbidly obese candidates for gastric bypass surgery within the Emam Reza Hospital. After fulfilling the informed consent, medical assessments were performed. Patients were included according to the following criteria: body mass index (BMI) higher than 40 kg/m^2^ or 35 kg/m^2^ with more than two comorbidities, no more than 30 g/day and 20 g/day alcohol intake in males and females, respectively, no liver damage due to medication, negativity of HBs antigen and HCV antibody. All procedures were approved by the local Ethical Committee and were in line with the ethical standards of the institutional research committee and with the 1964 Helsinki Declaration and its later amendments or comparable ethical standards.

## 3. Laboratory Assessment

Morning blood samples were obtained from each patient after a 12 h fast. Blood samples were centrifuged at 10,000× *g* for 15 min, and the serum separated. Laboratory assessment including lipid panel, glycemic status (fasting blood sugar (mg/dL), fasting insulin (μIU/L), Homeostasis Model Assessment Insulin Resistance (HOMA-IR) index, liver enzymes (alkaline phosphatase (ALP, U/L), aspartate aminotransferase (AST, U/L), gamma-glutamyl transferase (GGT, U/L) alanine aminotransferase (ALT, U/L), and inflammation status (hs-CRP (mg/L) were performed by photometric assay with the use of a biochemistry autoanalyzer (Alfa-Classic; Tajhizat Sanjesh Co., Ltd., Isfahan, Iran) and commercial kits (Pars Azmoun kit, Tehran, Iran).

## 4. Anthropometric Indices

Anthropometric indices were assessed using a standard protocol including height, waist circumference, weight (in light clothing and barefoot). Body composition (body fat mass and percentage, and body fat-free mass and percentage) were measured by bioelectrical impedance analyzer, Tanita BC-418 (Tanita Corp., Tokyo, Japan).

## 5. Definition

Type 2 diabetes mellitus was diagnosed in the presence of fasting blood glucose (FPG) ≥ 126 mg/dL or symptoms of hyperglycemia and a random plasma glucose ≥ 200 mg/dL or 2 h plasma glucose ≥ 200 mg/dL during an oral glucose tolerance test. Arterial hypertension is defined in the presence of a systolic blood pressure (SBP) ≥ 140 mmHg and/or a diastolic blood pressure (DBP) ≥ 90 mmHg by two measurements in the office or clinic on at least three different visits over a period of 3 to 6 months.

Impaired fasting glucose (IFG) was defined as fasting blood glucose (110–125 mg/dL) is an indicator of insulin resistance. The metabolic syndrome is defined in the presence of three or more of five criteria including: waist circumference > 102 cm (men) and >88 cm (women), blood pressure higher than 130/85 mmHg, fasting triglyceride (TG) levels ≥ 150 mg/dL, fasting high-density lipoprotein (HDL) cholesterol < 40 mg/dL (men) or <50 mg/dL (women) and fasting blood glucose ≥ 110 mg/dL according to the criteria of the National Cholesterol Education Program expert panel on detection, evaluation, and treatment of high blood cholesterol in adults (NCEP ATP III) [40].

## 6. Two-Dimensional Shear Wave Elastography and Ultrasonography

Liver stiffness and liver steatosis were measured using Two-dimensional shear-wave elastography (2D-SWE) in right lateral decubitus and abducted right arm position after six hour-fasting. The procedure was performed by Aixplorer ultrasound system (Supersonic Imagine, Aix-en-Provence, France) using curved broadband probe (SC6-1, 1–6 MHz). The mean of ten image acquisitions for each individual was considered as an optimal liver stiffness result. A single operator reported liver stiffness measurement (LSM) as the mean (M) of valid measurements in kilopascals (kPa); he was blinded to the patient data.

Grades of steatosis were classified as follows: S0, no steatosis (<5% hepatocytes); S1, mild (5–25% hepatocytes); S2, moderate (25–66% hepatocytes); and S3, severe (>66% hepatocytes).

Fibrosis-4 index (FIB 4) was defined based on the formula: age (years) × AST (U/L)/(platelets (109/L) × (ALT [U/L])1/2). Non-alcoholic fatty liver disease fibrosis score (NFS) definition was: −1.675 + 0.037 × age (years) + 0.094 × BMI (kg/m^2^) + 1.13 × IFG/diabetes (yes = 1, no = 0) + 0.99 × AST/ALT ratio−0.013 × platelet count (×109/L)−0.66 × albumin (g/dL).

## 7. Gastric Bypass Surgery

An ante-colic and ante-gastric Roux-en-Y gastric bypass was performed which resulted in a roux limb (100–150 cm) and a short biliopancreatic limb (nearly 70 cm). Consequently, a vertically oriented gastric pouch remained (30–50 cc). End-to-side gastrojejunostomy and side-to-side jejunojejunostomy were created by a three-row stapler. All the biopsy samples were obtained intraoperatively under direct visualization using a 16-gauge Tru-Cut needle extraction of the left hepatic lobe.

## 8. Post-Surgery Follow-up

Six months after surgery, all the patients were submitted to the same assessments, including anthropometric characteristics (weight, waist circumference, fat mass and fat-free mass percentage), blood concentrations of metabolic, liver, and inflammatory parameters as well as liver elastography.

## 9. Success Rate

Successful weight loss was considered as an excess weight loss (EWL) > 50% six months after gastric bypass surgery. The percentage of EWL (%EWL) was calculated using the formula: %EWL = ((pre-bariatric surgery weight minus weight at the time of visit)/(pre-bariatric surgery weight minus ideal weight)) × 100. The percentage of total weight loss (%TWL) was defined as: pre-operative weight minus the follow-up weight divided by the pre-operative weight and multiplied by 100. The percentage of excess BMI loss (%EBMIL) was estimated as [(PreoperativeBMI − currentBMI)/(preoperativeBMI − 25)] × 10 [41].

## 10. Statistical Analyses

Data were described as mean (standard deviation [SD]) and median (interquartile range [IQR]) for parametric and non-parametric variables, respectively. The associations between data were assessed using Spearman’s correlation coefficients. Regression models were used to assess the relationships between log-hs-CRP (dependent variable) and post-surgery fatty liver disease and success rate (independent variables). A 5% *p*-value was considered the significance cutoff (SPSS, version 25).

## 11. Results

### 11.1. Demographic Data

Among the 90 patients who were included in the study, 72 (80%) were females; mean age, weight, and BMI were 38.5 ± 11.1 years, 121.34 ± 20.32 kg, and 45.46 ± 6.26 kg/m^2^, respectively. More than half (51.9%) of the participants had the metabolic syndrome. Mean pre-operative liver stiffness was 6.1 ± 1.25 kPa. Postoperative complications were reported in 6 patients (1 pulmonary embolism, 1 Pulmonary edema, 1 short bowel syndrome, 1 abcess and 2 gastrointestinal bleeding) (Table 1).

### 11.2. Anthropometric Indices before and after Surgery

**Figure 1:**
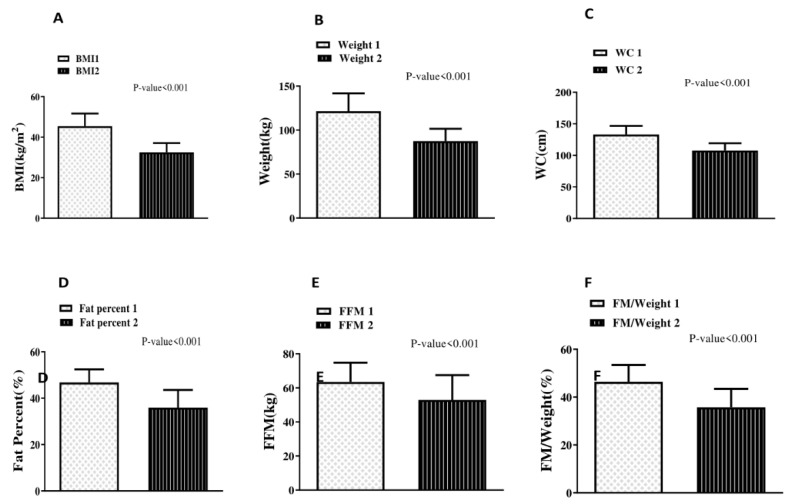
Anthropometric indices before and after bariatric surgery. BMI: body mass index; FFM: fat free mass; FM: fat mass; WC: waist circumference.

The average weight loss was 33.93 ± 11.79 kg (25.31 ± 9.40 kg and 11.88 ± 12.86 kg due to loss in fat and fat free mass, respectively). There was a significant decrease in all the anthropometric indices before and six months after surgery (*p* < 0.001) (Figure 1). Post-surgery, the mean %TWL was 27.96 ± 6.43%, ranging from 11.47% to 47.75%. On average, post-surgery %EBMIL and %EWL were 63.70 ± 15.27% and 63.92 ± 14.66%, respectively.

### 11.3. Liver and Inflammation Status before and after Surgery

On average, liver elasticity was significantly reduced from 6.1 ± 1.25 to 5.42 ± 1.52 kPa (*p* = 0.002). Liver aminotransferases decreased, reaching a significant difference for ALT, GGT, and ALP (*p* < 0.001). NFS and the grade of steatosis significantly reduced too (Table 2).

Platelet count was reduced by 22.96 ± 60.45 (number/μL) (*p* < 0.001). Serum hs-CRP levels reached a significant reduction (from 9.26 ± 8.45 to 3.29 ± 4.41 mg/L) (Table 2).

### 11.4. The Relationship between hs-CRP Levels and Liver Status after Bariatric Surgery

The relationships between pre-surgery hs-CRP and liver fibrosis (elastography), steatosis (ultrasonography), FIB-4, NFS, and surgery success rate are presented in Table 3. The correlations between hs-CRP levels and liver fibrosis (elastography), steatosis (ultrasonography), FIB-4, NFS, and surgery success rate were not significant.

### 11.5. Association between Baseline hs-CRP and Post-Surgery Success Rate and Liver Status at Regression Analyses

Binary logistic regression analyses were run to assess the association between baseline hs-CRP and post-surgery success rate and fibrosis (elastography); ordinal regression analyses were performed to study the relationship between baseline hs-CRP and post-surgery steatosis (ultrasonography) and linear regression analyses estimated the association between baseline hs-CRP and post-surgery FIB-4 index. All the models were adjusted for age, sex, baseline waist circumference, AST, ALT, GGT, ALP, and HOMA-IR values. A linear regression analysis assessed the association between hs-CRP and post-surgery NFS after adjusting for baseline sex, GGT, and ALP (Table 4). Regression analyses showed that serum hs-CRP levels were not predictive of liver status and success rate after surgery in both unadjusted and adjusted models.

## 12. Discussion

A significant reduction in hs-CRP levels, liver stiffness and steatosis occurred in patients with severe obesity at 6 months after bariatric surgery. Baseline hs-CRP values were associated neither with the weight loss success rate nor with post-surgery liver status.

Obesity is associated with other deleterious metabolic conditions including a chronic sub-clinic inflammatory status [42]. By the same token, elevated hs-CRP levels and hepatic steatosis are reported more frequently in metabolicaly healthy obese group compared to metabolicaly healthy normal weight group [43]. Our patients showed at baseline increased hs-CRP levels consistently with other studies [44]. CRP is a strong independent predictor of cardio-metabolic events, being implicated in the atherosclerotic process by modulation of endothelial function [45,46,47]. In addition, it increases insulin resistance by acting on insulin signaling through IRS-1 phosphorylation, thus impairing effective translocation of glucose transporter 4 (GLUT4) and insulin-stimulated glucose uptake [48,49]. Increased hs-CRP concentrations have been associated with NAFLD [43,50], and elevated levels of hs-CRP are related to the severity of the fatty liver disease [50]. However, the causal role of inflammation in NAFLD pathophysiology and its progression has not been clearly elucidated [50]. We need to identify good biomarkers to predict outcome after bariatric surgery using biological system models.

### 12.1. Hs-CRP Changes after Bariatric Surgery

A significant reduction in hs-CRP levels as early as 6 months after gastric bypass was evident in our patients. This reduction is in line with the results of previous studies [20,21,22,23,24,25,26,27,28,29,30] which reported a reduction ranging from 29% [26] to around 90% [27] in the levels of CRP after bariatric surgery. A direct relationship between the degree of weight loss and CRP reduction was observed in several studies [20,24], but not all [51].

Although the anti-inflammatory mechanisms of bariatric surgery remain mostly unknown, the post-surgery reduction in body visceral fat and adipocyte mass have been hypothesized as one of the main responsible factors in the reduction of inflammation [52]. Indeed, adipocytes release proinflammatory cytokines, such as TNF-α and its soluble receptors (sTNFR1, sTNFR2), and IL-6, both stimulating hepatocytes to produce acute-phase proteins, including CRP [7,44].

### 12.2. Liver Status Changes after Bariatric Surgery

Liver enzymes and ultrasonographic steatosis and elastographic fibrosis significantly improved in our patients at 6 months after bariatric surgery. The best preventive strategy for NAFLD is modifying the risk factors [53]. Bariatric surgery consistently ameliorated both biochemical and histological markers of NAFLD in several studies [54,55,56,57,58]. A recent metanalysis reported the improvement or resolution of liver fibrosis in 30% of patients after bariatric surgery, and Roux-en-Y gastric bypass seems to have the greatest impact on the NAFLD histology when compared with the other procedures [59]. The mechanisms potentially involved are: the increase in insulin sensitivity; the reduction in the adipose tissue lipolysis rate; the decrement of the endogenous glucose production and hepatic VLDL-triglycerides secretion; and the lowering of the hepatic expression of several factors involved in hepatic inflammation (MCP-1 and IL-8) and fibrogenesis (TGF-β1, TIMP-1, α-SMA, collagen-α1) [56]. In addition, bariatric surgery leads to increased adiponectin plasma levels [13,60], an adipokine with a protective effect towards the progression of NAFLD due to its anti-inflammatory and insulin sensitizing properties [61].

Our data showing a significant decrease in liver aminotransferases and in the grade of steatosis and stiffness confirmed the role of bariatric surgery as an option for the treatment of NAFLD in patients with obesity.

### 12.3. Hs-CRP and Weight Loss after Bariatric Surgery

We failed to find an association between pre-surgery hs-CRP values and post-bariatric weight loss. Differently form the well-known adverse predictive value of the post-bariatric CRP rise on surgical outcomes [33,34,62,63,64], few contrasting data about pre-surgery hs-CRP values on these outcomes are available. Considering costs and risks of bariatric surgery, the knowledge of the conditions predicting its success rate would lead to a better selection of the candidates mostly taking benefit from the intervention. It was previously reported that patients with a higher degree of inflammation better responded to bariatric surgery [38,39]. In a small study on 32 patients, those with higher baseline CRP values showed increased rates of T2DM remission after 3 year from surgical intervention and had a longer disease-free period [39]. In a larger study, high baseline levels of hs-CRP were able to predict increased reduction in visceral adipose fat 1 year after sleeve gastrectomy, independently of BMI reduction [38]. Furthermore, the higher the baseline CRP values, the greater was their decrease after surgery and patients with lower baseline values of hs-CRP experienced a subsequent increase after intervention [38]. The opposite association was found in 105 patients undergoing laparoscopic Roux-en-Y gastric bypass, i.e., lower pre-bariatric CRP levels predicted greater weight loss after 2 years [37]. However, in a multivariate regression model, the association with CRP was no longer significant [37].

Data on the predictive role of baseline CRP values are therefore controversial; the different surgical methods and the small samples size contribute to making the interpretation of literature difficult. The lack of association between pre-surgery hs-CRP and post-surgery weight loss could be due to the short follow-up time. Since maximal weight loss is achieved 1 or 2 years after surgery [65], it is not possible to exclude a role of CRP in predicting weight loss over a longer time-period.

### 12.4. Hs-CRP and Liver Status after Bariatric Surgery

In our patients, serum pre-surgery hs-CRP levels were not associated either with liver enzymes or ultrasonographic steatosis/elastographic fibrosis. This is the first study that investigated the role of pre-bariatric hs-CRP in predicting the improvement in liver status after surgery.

Patients with liver steatosis showed increased hs-CRP levels independent of their BMI [66] and hs-CRP was reported as an obesity-independent marker of NAFLD severity [67,68]. It is documented that elevated hs-CRP values even within the normal range are associated with higher risk of NAFLD development particularly in the presence of hepatic steatosis. Thus, high-normal hs-CRP levels within healthy individuals could serve as a predictor of NAFLD and warrant a close follow-up to avoid further complications [69]. Therefore, we can speculate that pre-bariatric hs-CRP values do not predict whether liver disease will improve as a result of the post-surgery weight loss since hs-CRP and NAFLD association is independent from weight. The association between CRP and steatosis is likely to be complex, and factors other than weight status might also contribute. Consequently, hsCRP is a favorable predictor of disease progression in patients undergoing bariartric surgery and also in atypical individuals who have unnoticeable clinical and laboratory findings [70]. It could be hypothesized that the before-after change in CRP levels could be a better predictor of liver status variations after surgery rather than pre-surgical CRP value. Again, due to the short follow-up of our study, the possibility that a long-term evaluation may provide different results can not be totally excluded.

## 13. Limitations

The short follow-up and the small sample size are limitations of the present study. Although a greater weight loss could be observed only after a longer period of follow-up, it has been reported that a greater early weight loss is a strong predictor of long-term outcomes [71]. A liver biopsy has not been performed; indeed, elastography and ultrasonography are non-invasive validated methods to estimate the liver stiffness or the extent of steatosis, respectively, in patients with liver disease [72]. Despite a comprehensive screening prior to surgery such as evaluation of a malignancy and autoimmune diseases, some potential inflammatory diseases such as periodontitis were not examined.

## 14. Conclusions

The results of our study confirm that bariatric surgery is effective in reducing inflammatory state and improving liver stiffness and steatosis in patients with morbid obesity. Baseline values of hs-CRP were not be able to predict weight loss and liver status after surgery. However, further studies with a longer follow-up are needed to elucidate the complex relationship between pre-bariatric inflammatory status and post-surgery outcomes. The application of systems biology methods, such as proteomic study will enable the identification of suitable biomarkers.

## Figures and Tables

**Table 1:** Patient Characteristics.

Variable	Total
Male (%)	18 (20)
Age (years)	38.5 ± 11.1
BMI (kg/m^2^)	45.46 ± 6.26
Weight (kg)	121.34 ± 20.32
Waist Circumference (cm)	133.04 ± 13.6
Height (m)	1.62 ± 8.87
Type 2 diabetes mellitus (%)	25 (27.8)
Arterial hypertension (%)	23 (25.6)
Metabolic syndrome (%)	46 (51.1)

BMI; body mass index.

**Table 2:** Liver and inflammation status before and after bariatric surgery.

Variable	Before	After	*p*-Value
Liver stiffness measurement, kPa	6.10 ± 1.25	5.42 ± 1.52	0.002
AST (IU/L)	21 (17.00; 29.00)	19 (16.00; 25.00)	0.027
ALT (IU/L)	25 (17.00; 38.50)	16.5 (12.00; 25.00)	<0.001
GGT (IU/L)	27 (20.00; 34.50)	14 (11.00; 19.00)	<0.001
ALP (IU/L)	196.25 ± 53.79	222.50 ± 65.61	<0.001
Platelets (number/μL)	303.43 ± 71.20	280.47 ± 66.12	<0.001
FIB 4	0.53 (0.37; 0.73)	0.66 (0.43; 0.94)	<0.001
NFS	−1.35 (−2.44; −0.39)	−2.40 (−3.16; −1.41)	<0.001
Steatosis (ultrasonography) (%)			<0.001
Grade 0	5 (5.5)	16 (18)	
Grade 1	19 (21.1)	47 (52.8)	
Grade 2	53 (58.8)	24 (27)	
Grade 3	13 (14.4)	2 (2.2)	
Hs-CRP (mg/L)	5.05 (2.40; 13.60)	1.60 (0.80; 4.02)	<0.001

Mean ± SD or median (95% CI); ALT: alanine aminotransferase; AST: aspartate aminotransferase; FIB4: fibrosis 4; GGT: gamma glutamyl transferase; hs-CRP: high-sensitive C reactive protein; NFS: NAFLD fibrosis score.

**Table 3:** Relationship between baseline hs-CRP and liver status after bariatric surgery.

	hs-CRP (mg/L)	
CC	Rho	*p*-Value
FIB-4	0.057	0.657
NFS	0.002	0.985
Fibrosis (elastography)	0.045	0.704
Steatosis (ultrasonography)	0.164	0.154

hs-CRP: high-sensitivity C-reactive protein; CC: correlation coefficient; FIB4: fibrosis 4; NFS: NAFLD fibrosis score; Rho: Spearman’s correlation coefficient.

**Table 4:** Association between baseline hs-CRP levels and liver status and success rate after surgery.

Parameters		*p*	OR	95% CI for OR	
				Lower	Upper
Crude Model	FIB4	0.914	−0.001	−0.013	0.012
	NFS	0.967	0.001	−0.041	0.043
	Success rate	0.829	0.994	0.940	1.050
	Fibrosis (Elastography)	0.688	1.014	0.947	1.085
	Steatosis (Sonography)	0.077	1.05	0.99	1.11
Adjusted Model	FIB4 *	0.866	−0.018	−0.237	0.200
	NFS **	0.442	0.254	−0.403	0.912
	Success rate *	0.479	1.466	0.508	4.226
	Fibrosis * (Elastography)	0.389	−0.382	−1.264	0.499
	Steatosis * (Sonography)	0.674	0.206	0.753	1.165

Log-transformed hs-CRP values were used. Logistic regression analysis for log-hs-CRP and success rate. Linear regression analysis for log-hs-CRP and FIB4, NFS and Fibrosis. Ordinal regression analysis for log-hs-CRP and Steatosis. * Model adjusted for sex, waist circumference, HOMA-IR, GGT, ALP. ** Model adjusted for sex, GGT, ALP.

## Data Availability

Not applicable.
